# A comparison of methods for inferring causal relationships between genotype and phenotype using additional biological measurements

**DOI:** 10.1002/gepi.22061

**Published:** 2017-07-10

**Authors:** Holly F. Ainsworth, So‐Youn Shin, Heather J. Cordell

**Affiliations:** ^1^ Institute of Genetic Medicine Newcastle University, International Centre for Life, Central Parkway Newcastle upon Tyne United Kingdom; ^2^ MRC Integrative Epidemiology Unit (IEU) University of Bristol Bristol United Kingdom

**Keywords:** Bayesian networks, causal inference, Mendelian randomisation, structural equation modelling

## Abstract

Genome wide association studies (GWAS) have been very successful over the last decade at identifying genetic variants associated with disease phenotypes. However, interpretation of the results obtained can be challenging. Incorporation of further relevant biological measurements (e.g. ‘omics’ data) measured in the same individuals for whom we have genotype and phenotype data may help us to learn more about the mechanism and pathways through which causal genetic variants affect disease. We review various methods for causal inference that can be used for assessing the relationships between genetic variables, other biological measures, and phenotypic outcome, and present a simulation study assessing the performance of the methods under different conditions. In general, the methods we considered did well at inferring the causal structure for data simulated under simple scenarios. However, the presence of an unknown and unmeasured common environmental effect could lead to spurious inferences, with the methods we considered displaying varying degrees of robustness to this confounder. The use of causal inference techniques to integrate omics and GWAS data has the potential to improve biological understanding of the pathways leading to disease. Our study demonstrates the suitability of various methods for performing causal inference under several biologically plausible scenarios.

## 1 INTRODUCTION

Many genetic variants associated with human diseases have been successfully identified using genome wide association studies (GWAS) (Visscher, Brown, McCarthy, & Yang, 2012). However, a typical GWAS provides limited further insight into the biological mechanism through which these genetic variants are implicated in disease. The variants implicated by GWAS are not necessarily true causal variants (that directly influence disease risk) but may rather correspond to variants in linkage disequilibrium with the causal variant(s). Even for putative causal variants, there is typically a lack of understanding of how the identified genetic variants influence the phenotype at a molecular/cellular level. Consequently, moving towards therapeutic intervention is not straightforward.

It has become popular to use data from publicly available databases to provide functional evidence for loci that have been identified through GWAS (Cordell et al., 2015; Wain et al., 2017; Warren et al., 2017). For example, it may be of interest to consider whether a single nucleotide polymorphism (SNP) associated with disease associates with gene expression in a relevant tissue. If such an association can be demonstrated, it might indicate that the observed association between the SNP and disease phenotype is mediated through altering the level of gene expression. However, the individuals contributing to public databases are typically different from those who feature in the original GWAS data set (and the results may even derive from experiments on a different organism), making direct conclusions about causality problematic. We therefore consider instead the situation whereby we have measurements of a potential intermediate phenotype (such as gene expression) taken in the *same* set of individuals as are included in the GWAS data set. Use of such ‘overlapping’ sets of measurements allows us to address directly questions regarding the causal relationships between variables. This approach has been employed previously for examining the potential role of DNA methylation as a mediator between SNP genotype and rheumatoid arthritis (Liu et al., 2013) or ovarian cancer (Koestler et al., 2014), and for investigating the role of metabolites as a potential mediator between SNP genotype and various lipid traits (Shin et al., 2014).

In these previous studies, a filtering step based on consideration of pairwise correlations/associations between variables of different types was first used in order to filter the number of variables considered to a manageable level, retaining only those variables whose pairwise correlations reached a specified level of significance. All resulting ‘triplets’ of variables (consisting of a genetic variable, a potential mediator variable such as a variable related to DNA methylation or metabolite concentration, and an outcome variable such as rheumatoid arthritis or a lipid trait) were then subjected to a causal inference test (CIT)—the CIT (Millstein, 2016; Millstein, Zhang, Zhu, & Schadt, 2009) in Liu et al. (2013), and Mendelian randomisation (Smith & Ebrahim, 2003) and structural equation modelling (Bollen, 1989) in Shin et al. (2014)—in order to elucidate the causal relationships between the variables in each triplet. Use of a similar pairwise filtering approach was employed by Zhu et al. (2016), who developed a method known as SMR (summary data‐based Mendelian randomisation). SMR uses GWAS summary statistics (SNP effects) together with eQTL summary statistics from publicly available databases to test for association between predicted gene expression and phenotype, with a further test known as HEIDI (heterogeneity in dependent instruments) used to elucidate causal relationships between triplets of variables; in their application Zhu et al. (2016) restricted the HEIDI analysis to expression probes that (a) showed association at $P<5\times 10^{-8}$ with nearby SNPs (so‐called cis‐eQTLs) and (b) also showed association at $P<8.4\times 10^{-6}$ with one of five complex traits considered. In an expanded version of this study, Pavlides et al. (2016) increased the number of phenotypes considered to 28 complex traits and diseases, while using the same filtering thresholds to focus the HEIDI analysis on 271 triplets of variables, each consisting of a SNP (cis‐eQTL), its associated gene expression probe and a complex trait with which the gene expression probe is also associated.

More ambitiously, the (probabilistic) construction of entire causal networks of multiple variables, including metabolomic and transcriptomic (gene‐expression) measurements, has been carried out using approaches based on Bayesian networks (Zhu et al., 2004, 2012). This approach allows in principle the simultaneous consideration of a potentially large number of variables. Bayesian networks can only be solved at the level of Markov (mathematically) equivalent structures; however genetic data can be incorporated in the network prior as ‘causal anchor’ to help direct the edges in the network. Although the Bayesian networks considered generally contain large numbers of variables, this incorporation of genetic data in order to help direct edges has typically involved calculations performed on smaller subunits such as triplets of variables (e.g., one genetic factor and a pair of nongenetic factors such as metabolite concentrations or gene expression values)  (Zhu et al., 2004, 2012). The use of genetic data as a causal anchor for delineating the causal relationships between other variables (in particular between modifiable risk factors and phenotypic outcome) has a long history in the field of genetic epidemiology and has been popularised in the approach of Mendelian randomisation (Smith & Ebrahim, 2003) and its extensions (such as SMR, described above).

Given the focus, thus far, in the literature, on using triplets of variables to perform causal inference, we were interested to examine the performance of the available methods in this simple situation, before moving to the more complex situation of analysing multiple variables (as are routinely encountered in modern ‘omics’ data sets) simultaneously. We chose to investigate the following methods for causal inference: Mendelian randomisation (Smith & Ebrahim, 2003), a CIT (Millstein, 2016; Millstein et al., 2009), structural equation modelling and several Bayesian methods. We present a simulation study that assesses the performance of the methods under different conditions, assuming throughout that we have genotype data along with two observed quantitative (continuous) phenotypes. We also consider how inference is affected by the presence of unmeasured environmental confounding factors. We begin by outlining the details of our simulation study before presenting an overview and discussion of the results.

## 2 METHODS

For the purposes of our study, we assume we have genotype data (*G*) from a single SNP, along with measurements of gene expression (*X*) and a further phenotype of interest (*Y*). In reality, *X* could be any omics measurement of interest (e.g., gene expression, DNA methylation, metabolite concentration, proteomic measurements etc.). We assume that it is known that there exist some pairwise associations between the variables; this could have been established during a preprocessing or filtering step.

**Figure 1:**
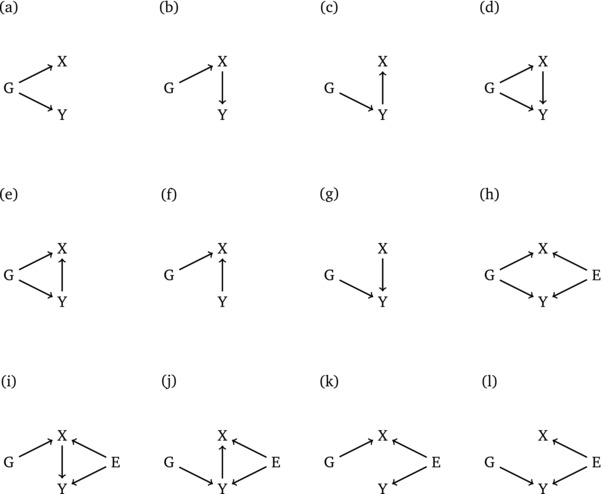
Possible causal models explaining the relationship between a genetic variant *G* and two observed traits *X* and *Y*. Models (h)–(l) include an unmeasured common enviromental effect *E*

Figure 1 shows some hypothesised causal models to explain the relationship between the variables *G*, *X*, and *Y*. Where an arrow is present between two variables, this is indicative of a causal relationship between these variables, the direction is characterised by the direction of the arrow. The set of models is restricted to those that are biologically plausible, consequently we do not consider models in which the genetic variant *G* can be influenced by any other variable. In models (h)–(l), we also include an unmeasured confounder corresponding to an environmental effect *E*.

Given observed data on *G*, *X*, and *Y*, we were interested to explore how well the underlying causal structure can be learned. We consider several commonly used techniques for attempting to infer underlying causal structure between variables. We first consider two methods designed to detect causal associations in specific scenarios: Mendelian randomisation (MR) (Smith & Ebrahim, 2003) and a CIT (Millstein, 2016; Millstein et al., 2009). These methods are not designed for an exploratory analysis involving many structures and would normally only be used when there is a strong prior hypothesis that a particular causal model gave rise to the data. Nevertheless, we consider it useful to explore how well these methods perform on our simulated data sets. We also consider several approaches used for causal modelling that are more flexible, these are structural equation modelling (SEM) (Bollen, 1989; Fox, Nie, & Byrnes, 2015), a Bayesian unified framework (BUF) (Stephens, 2013), and two different R packages for learning Bayesian networks: DEAL (Bottcher & Dethlefsen, 2013) and BNLEARN (Scutari, 2010). A more detailed overview of all of these techniques is provided in the Supporting Information.

### 2.1 Simulation Study

For each of the 12 causal scenarios given in Figure 1, 1,000 replicate data sets were simulated, each containing 1,000 individuals. The SNP genotype data (*G*) were generated assuming Hardy‐Weinberg equilibrium and a minor allele frequency of 0.1. The direct effect sizes were initially chosen to be constant throughout all models. For example, when simulating data from model (a) in Figure 1, the effect size of *G* on *X* is the same as the effect size of *G* on *Y*. Full details of the simulation models are given in Table 1. For each simulated data set, we applied each of the six causal inference methods under consideration. The idea was to assess how well these methods could recover the true underlying causal structure. Because the methods we consider approach the problem from different angles, direct comparison of results is not straightforward. MR and the CIT are designed to test for specific causal scenarios, usually informed by prior knowledge. In our setup, MR is designed to identify the causal relationship X→Y while the CIT identifies that *X* acts as a mediator between *G* and *Y* (i.e., identifies the relationship G→X→Y) and, moreover, that *X* is the only causal link between *G* and *Y*. For MR and the CIT, we consider that the specified causal relationships have been established if a significant *P*‐value (P<0.05) is returned from the respective test.

**Table 1:** Details of simulation models for scenarios given in Figure 1

	Simulation model		
Scenario	*X*	*Y*	*E*
(a)	$X|G\;∼\;N(\mu_{X}+\alpha G,\;\sigma_{X}^{2})$	$Y|G\;∼\;N(\mu_{Y}+\beta G,\;\sigma_{Y}^{2})$	
(b)	$X|G\;∼\;N(\mu_{X}+\alpha G,\;\sigma_{X}^{2})$	$Y|X\;∼\;N(\mu_{Y}+\gamma X,\;\sigma_{Y}^{2})$	
(c)	$X|Y\;∼\;N(\mu_{X}+\gamma Y,\;\sigma_{X}^{2})$	$Y|G\;∼\;N(\mu_{Y}+\beta G,\;\sigma_{Y}^{2})$	
(d)	$X|G\;∼\;N(\mu_{X}+\alpha G,\;\sigma_{X}^{2})$	$Y|G,\;X\;∼\;N(\mu_{Y}+\beta G\;+\gamma X,\;\sigma_{Y}^{2})$	
(e)	$X|G,\;Y\;∼\;N(\mu_{X}+\alpha G+\delta Y,\;\sigma_{X}^{2})$	$Y|G\;∼\;N(\mu_{Y}+\beta G,\;\sigma_{Y}^{2})$	
(f)	$X|G,\;Y\;∼\;N(\mu_{X}+\alpha G+\delta Y,\;\sigma_{X}^{2})$	$Y\;∼\;N(\mu_{Y},\;\sigma_{Y}^{2})$	
(g)	$X\;∼\;N(\mu_{X},\;\sigma_{X}^{2})$	$Y|G,\;X\;∼\;N(\mu_{Y}+\beta G\;+\gamma X,\;\sigma_{Y}^{2})$	
(h)	$X|G,\;E\;∼\;N(\mu_{X}+\alpha G+\zeta E,\;\sigma_{X}^{2})$	$Y|G,\;E\;∼\;N(\mu_{Y}+\beta G+\zeta E,\;\sigma_{Y}^{2})$	$E\;∼\;N(0,\sigma_{E}^{2})$
(i)	$X|G,\;E\;∼\;N(\mu_{X}+\alpha G+\zeta E,\;\sigma_{X}^{2})$	$Y|X,\;E\;∼\;N(\mu_{Y}+\gamma X+\zeta E,\;\sigma_{Y}^{2})$	$E\;∼\;N(0,\sigma_{E}^{2})$
(j)	$X|Y,\;E\;∼\;N(\mu_{X}+\delta Y+\zeta E,\;\sigma_{X}^{2})$	$Y|G,\;E\;∼\;N(\mu_{Y}+\beta G+\zeta E,\;\sigma_{Y}^{2})$	$E\;∼\;N(0,\sigma_{E}^{2})$
(k)	$X|G,\;E\;∼\;N(\mu_{X}+\alpha G+\zeta E,\;\sigma_{X}^{2})$	$Y|E\;∼\;N(\mu_{Y}+\zeta E,\;\sigma_{Y}^{2})$	$E\;∼\;N(0,\sigma_{E}^{2})$
(l)	$X|E\;∼\;N(\mu_{X}+\zeta E,\;\sigma_{X}^{2})$	$Y|G,\;E\;∼\;N(\mu_{Y}+\beta G\;+\zeta E,\;\sigma_{Y}^{2})$	$E\;∼\;N(0,\sigma_{E}^{2})$

The default parameter values are α= 1, β= 1, δ= 1, $\mu_{X}$= 10, $\mu_{Y}$= 10, γ= 1, ζ= 1, $\sigma_{X}$= 0.3, $\sigma_{Y}$= 0.3, $\sigma_{E}$= 0.3. *G* is coded as (0, 1, 2) according to the number of minor alleles present at the SNP

The other four methods are more flexible because they all consider a wider range of causal models. The Bayesian network methods (DEAL and BNLEARN) can consider the full space of models arising from three variables, including models (a)–(g) in Figure 1. However, they naturally exclude any models with an arrow going towards the SNP because the methods assume that discrete variables do not have continuous parents. This convenient feature of Bayesian networks automatically imposes the natural biological assumption that genetic factors (such as SNPs) are assigned at birth and will not be influenced by any other of the measured variables. The Bayesian network methods assign to each model a network score, and we consider the model with the highest network score to be the most plausible.

For SEM, not all structures are considered as only a subset of models have enough degrees of freedom to be testable. These models are (a), (b), (c), (f) and (g) from Figure 1. We choose the model with the lowest Bayesian information criterion (BIC) (Schwarz, 1978) to be the most plausible. The BUF method considers all possible partitions of variables *X* and *Y* into three categories: *U* (unassociated with *G*), *D* (directly associated with *G*), and *I* (indirectly associated with *G*). This gives a total of nine partitions. Of these nine partitions, three correspond to models in Figure 1, namely (a), (b), and (c). In the following, we will refer to two further partitions, (m) and (n), where (m) represents a model with just one arrow G→X and (n) represents a similar model with G→Y. We take the model with the highest Bayes factor to be the most plausible.

## 3 RESULTS

**Figure 2:**
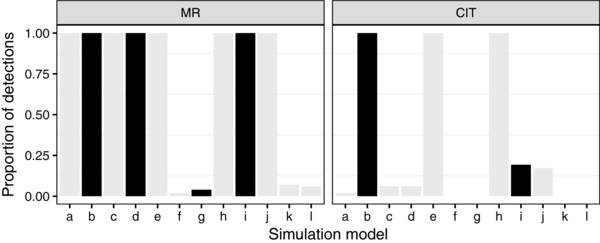
Results of applying MR and the CIT to simulated data sets. The *x*‐axis represents the scenario from which the data were simulated. The *y*‐axis represents the proportion of time (the proportion of replicates where) a causal model was detected (X→Y for MR, and G→X→Y with *X* the only link between *G* and *Y*, for the CIT). Black and grey represent *true* and *false* detections, respectively. For MR, we considered detections from simulated data sets with an arrow X→Y as *true* detections. For the CIT, we considered detections from simulated data sets with arrows G→X→Y but no additional link between *G* and *Y* as *true* detections

Figure 2 shows the results of applying MR and the CIT to simulated data sets. In each plot, the *x*‐axis indicates the scenario under which the data have been simulated, as illustrated in Figure 1. The *y*‐axis represents the proportion of simulated data sets in which the test detects a specified causal relationship. This relationship is X→Y for MR and G→X→Y (with no other causal link between *G* and *Y*) for the CIT.

As expected, for data simulated under scenario (b), the causal structure can be successfully identified (as highlighted in black) by both methods. It is also of interest to consider how the methods perform for data simulated under scenario (i), which is akin to model (b) with the addition of an unmeasured common environmental effect. MR was able to successfully suggest a causal relationship X→Y existed in scenario (i), whereas the CIT did not typically establish the causal structure G→X→Y (with no other causal link between *G* and *Y*). For data simulated under other scenarios, both methods incorrectly identified the specified causal relationships some of the time (shown in grey). However, this is not unexpected because in these cases there has typically been a violation of the modelling assumptions.

In these initial simulation scenarios, both MR and CIT performed well when their assumptions were satisfied, with the existence of a causal link between *X* and *Y* identified 100% of the time under scenario (b) (Fig. 2). However, one might expect that the performance of both methods would deteriorate when the relationships between the variables (either between *G* and *X* or between *X* and *Y*) are less strong. Supporting Information Figure S1 shows the results of lowering either the effect size of *G* on *X* (α) or the effect size of *X* on *Y* (γ), while keeping all other effects constant, for data simulated under scenario (b). When α or γ are sufficiently low (<0.012), encapsulating the situation of much weaker relationships between the variables, we find that performance does indeed deteriorate, with MR achieving overall higher power than the CIT in this situation.

The results of causal inference using SEM, BUF, DEAL, and BNLEARN are shown in Supporting Information Figures S2– S5 and summarized numerically in Table 2. In this table, each cell represents an average score calculated from the 1,000 replicate data sets. Columns represent data simulated under the 12 different scenarios in Figure 1 and rows describe which model is being tested. For each of the four methods of inference, a different score is calculated. For SEM, we use BIC and models with low BIC scores are considered to be a better fit. For the other three methods, the better fitting models have the higher numeric scores assigned to them. The model(s) that are considered on average most likely (i.e., that have the lowest average BIC for SEM, or the highest average score for the other methods) are underlined. Where a cell is marked in bold, this highlights the correct model choice. For models (a)–(g), we consider the inferred model to be correct when the simulation model is recovered precisely. However, for models (h)–(l), we assume the correct model is the one which corresponds to the simulation model with the variable *E* omitted.

**Table 2:** Results from performing causal inference on simulated data sets

		Simulation model											
Method	Tested model	a	b	c	d	E	f	g	h	i	j	k	l
SEM	a	**−6**	689	688	689	689	686	685	**282**	1,381	1,380	282	283
	b	504	**−6**	402	503	− 6	399	1,088	148	**148**	553	148	841
	c	504	400	**−6**	− 6	505	1,091	398	148	552	**148**	840	148
	f	1,090	685	1,091	1,600	1,090	**−6**	684	688	282	687	− 6	686
	g	1,089	1,091	684	1,092	1,601	686	**−6**	687	686	282	687	− 6
BUF	a	**156.01**	120.93	120.7	155.97	156.18	120.84	121.06	**98.79**	98.84	98.95	98.80	98.78
	b	120.78	**120.95**	83.68	120.94	156.19	83.58	−0.08	83.61	**83.78**	37.89	83.76	−0.08
	c	120.95	83.64	**120.73**	155.98	121.13	−0.09	83.84	83.69	37.81	**83.90**	−0.08	83.71
	m	35.06	37.28	−0.03	−0.01	35.05	120.93	37.22	15.1	61.03	15.05	**98.88**	15.06
	n	35.23	−0.02	37.03	35.03	−0.01	37.25	121.14	15.18	15.07	61.06	15.04	**98.86**
DEAL	a	**−1,019**	−1,359	−1,360	−1,378	−1,379	−1,343	−1,343	**−1,697**	−2,245	−2,244	−1,689	−1,689
	b	−1,254	**−1,003**	−1,200	−1,264	− 1,019	−1,263	−1,530	−1,196	**−1,618**	−1,821	−1,620	−1,954
	c	−1,254	−1,199	**−1,004**	− 1,019	−1,263	−1,530	−1,196	−1,618	−1,821	**−1,625**	1,954	−1,619
	d	− 1,016	−1,011	−1,012	**−1,025**	−1,025	−1,010	−1,010	− 1,551	− 1,560	− 1,560	−1,553	−1,554
	e	− 1,016	−1,011	−1,012	−1,025	**−1,025**	−1,010	−1,010	− 1,551	− 1,560	− 1,560	−1,553	−1,554
	f	−1,541	−1,339	−1,537	−1,794	−1,550	**−1,004**	−1,339	−1,880	−1,693	−1,889	− 1,548	−1,884
	g	−1,541	−1,536	−1,341	−1,549	−1,793	1,338	**−1,005**	−1,880	−1,888	−1,693	−1,883	− 1,548
	m	−1,544	−1,688	−1,886	−2,148	−1,904	−1,338	−1,673	−2,027	−2,377	−2,573	**−1,684**	−2,019
	n	−1,544	−1,884	−1,690	−1,902	−2,147	−1,671	−1,338	−2,027	−2,573	−2,377	−2,019	**−1,683**
BNLEARN	a	**−976**	−1,322	−1,323	−1,323	−1,321	−1,322	−1,320	**−1,671**	−2,214	−2,215	−1,667	−1,668
	b	−1,230	**−973**	−1,178	−1,229	− 974	−1,176	−1,516	−1,601	**−1,596**	−1,799	−1,598	1,945
	c	−1,231	−1,176	**−975**	− 974	−1,228	−1,522	−1,173	−1,602	−1,799	**−1,597**	−1,944	−1,599
	d	−985	−984	−985	**−984**	−984	−984	−982	− 1,536	− 1,530	− 1,531	−1,531	−1,533
	e	−9,85	−984	−985	−984	**−984**	−984	−982	− 1,536	− 1,530	− 1,531	−1,531	−1,533
	f	− −1,530	−1,325	−1,531	−1,784	−1,528	**−979**	−1,320	−1,876	−1,669	−1,871	− 1,527	−1,874
	g	−1,532	−1,528	−1,328	−1,529	−1,782	−1,325	**−977**	−1,878	−1,872	−1,669	−1,873	− 1,528
	m	−1,521	−1,663	−1,869	−2,123	−1,864	−1,317	−1,658	−2,012	−2,353	−2,555	**−1,663**	−2,009
	n	−1,523	−1,866	−1,665	−1,867	−2,119	−1,663	−1,315	−2,013	−2,556	−2,353	−2,009	**−1,663**

Cells represent the average (over 1,000 replicates) of the scores describing how well each model fits the data. Columns represent data simulated under the 12 different scenarios and rows describe which model is being tested. Each of the four methods uses a different score to assess model fit. For SEM, low numeric scores indicate better fit. For the other three methods, higher numeric scores indicate better fit. Average score(s) that indicate the preferred model out of those tested are underlined. Cells with bold indicate the correct model choice.

For SEM, it can be seen that for data simulated the under scenarios that are testable, the correct model is identified as having the lowest BIC each time. Furthermore, the average BIC for the correct model is notably lower than that of its competitors. For scenario (h), SEM suggests that the most favourable model is either model (b) or (c). Here, the presence of an unmeasured/unknown environmental effect causes SEM to suggest that the effect of *G* is mediated by another variable rather than influencing *X* and *Y* independently. For data simulated under scenarios (i) and (j), SEM successfully suggests the best fitting models are (b) and (c), respectively. Although the other models are not directly testable, for data simulated under these scenarios, the inferences made by SEM appear largely sensible. For data simulated under models (k) and (l), models (f) and (g) are found to give the best fit, which seems reasonable as the causal link between *G* and *X* or *Y*, respectively, is retained. For data simulated under models (d) and (e) (which are Markov equivalent and therefore statistically indistinguishable), models (c) and (b), respectively, are inferred. This seems initially counter‐intuitive as the causal arrows between *X* and *Y* appear to have been inferred in the wrong direction. Our explanation for this is that, for data simulated under (d), the correlation between *G* and *Y* will be larger than the correlation between *G* and *X*, which better fits model (c) than it does models (a) or (b). Similarly, for data simulated under (e), the correlation between *G* and *X* will be larger than the correlation between *G* and *Y*, which better fits model (b) than it does models (a) or (c).

The results for BUF in Table 2 indicate that, when data are simulated from scenarios (a), (b), and (c), the correct models all have the highest average Bayes factor. When considering data with added environmental effects, BUF correctly identifies on average that data simulated under scenarios (k) and (l) come from scenarios (m) and (n). For scenario (h), BUF identifies the correct model, however, it fails to identify the correct model for scenarios (i) and (j). Models (d)–(g) are not testable by BUF, however, for data simulated under these scenarios, sensible models are chosen. It must be noted that many of the Bayes factors for competing models are very close in magnitude. In practice, it would not be sensible to favour one model over another on the basis of these Bayes factors alone. For example, the incorrect model has the highest Bayes factor under scenarios (b) and (c) in approximately 25% of data sets (see Supporting Information Fig. S3).

DEAL correctly identifies the correct model for data simulated under scenarios (b), (c), (f), and (g). However, for scenarios (a), (h), (i), and (j), DEAL suggests that models (d) or (e) are the most favourable. In each case, these models are overparameterised compared with the simulation model. This effect could be explained by the specification of the prior distribution in the DEAL method. The parameter ISS (imaginary sample size) governs how much weight is given to the prior distribution in the calculation of the network score and must be specified in any analysis which uses the DEAL method. Because there appears to be no consensus on how to choose this parameter, we initially used the default choice which for our data sets was ISS=6. The sensitivity of the network score to the choice of ISS has been previously documented (Silander, Kontkanen, & Myllymäki, 2007). We subsequently considered different choices of ISS and in Supporting Information Figure S6 we show that identification of the correct final model is indeed highly sensitive to the choice of ISS.

For BNLEARN, models (a)–(g) were testable and, for data simulated under these scenarios, the correct model gave the highest average network score in all cases apart from with data simulated under models (d) and (e). However the average network score for the correct model ((d) or (e), respectively) was not very different from that of the chosen model ((c) or (b), respectively). For data simulated under scenarios (h), (i), and (j), BNLEARN suggests that models (d) and (e) are the most likely. In these cases, the correct structure is identified but extra edges are suggested. For data simulated under scenarios (k) and (l), BNLEARN suggests that models (f) and (g) are most plausible and we consider these inferences to be sensible.

Statistically speaking, models (d) and (e) are indistinguishable. Both DEAL and BNLEARN make this fact clear by generating identical network scores for models (d) and (e), regardless of the input data. We consider this an appealing feature of these methods.

To assess the sensitivity of our study to the parameter choices used to simulate the data, we chose certain scenarios for further investigation. First, we considered changing the effect size ζ of the common environmental effect *E* in scenarios (h) and (i). Second, we considered changing α, which represents the effect size of *G* on *X*, in scenarios (a) and (b). In both cases we kept all other effect sizes the same.

**Figure 3:**
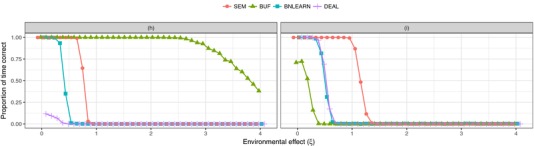
Results showing the effect of changing the effect size of the common environmental effect *E* (ζ) on inference. The *x*‐axis shows the value of ζ used and the *y*‐axis shows the proportion of time (the proportion of replicates where) the correct causal scenario was identified for data simulated under model (h) (left panel) and (i) (right panel)

Figure 3 displays the results of changing the effect size of the common environmental effect (ζ). In general, increasing the effect size of ζ results in a decreased proportion of correctly identified models. For scenario (h), BUF seemed to be able to infer the correct causal relationship the majority of the time, even when the effect size of *E* was around three times as large as other effect sizes. The other methods began to perform badly much sooner. For scenario (i), all methods were no longer able to correctly identify the correct causal model once the effect size for *E* reached around 1.5.

**Figure 4:**
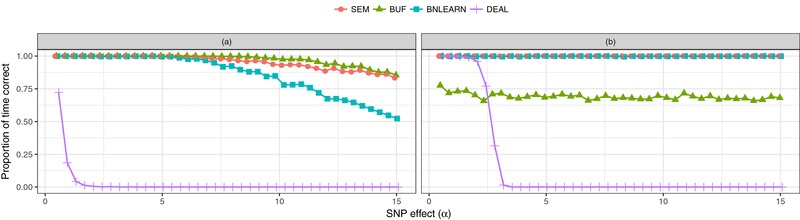
Results showing the effect of changing the G→X effect size (α) on inference. The *x*‐axis shows the value of α used in the simulation model, the *y*‐axis shows the proportion of time (the proportion of replicates where) the correct causal scenario was identified for data simulated under models (a) (left panel), (b) (right panel)

Figure 4 shows the results of changing the effect size of *G* on *X* (α) while keeping all other effects constant for data simulated under scenarios (a) and (b). This aims to replicate the very plausible biological scenario whereby the association between a SNP (*G*) and gene expression (*X*) is very strong but the association between gene expression and a phenotype (*Y*) is much weaker. In scenario (a), SEM, BUF, and BNLEARN all perform consistently well over a wide range of α values. For scenario (b), the accuracy of these three methods seems to be unaffected by the choice of α. The performance of DEAL in both scenarios seems particularly sensitive to the effect sizes considered.

## 4 DISCUSSION

Here, we have presented a simulation study considering the performance of a broad range of methods for inferring causal relationships when we have observed data on three variables: *G*, *X*, and *Y*. We envisaged a situation whereby these variables represent a genetic variant (*G*), a gene expression level (*X*) or other relevant biological measurement, and a phenotype of interest (*Y*). Several of the causal scenarios considered also included an unmeasured environmental effect (*E*), which modifies *X* and *Y*.

The methods that we considered for performing causal inference approach the problem from different perspectives. MR and the CIT assume an initial hypothesis regarding the structure of the causal effects and test this hypothesis accordingly, whereas the other four methods assume no such hypothesis but infer the most likely causal structure from data after enumerating all (or most) plausible structures. Although all methods—at least as implemented here—make use of essentially the same data (measurements of phenotypic outcome, genotypic exposure and potential intermediate biological variables or *mediators*), the use of SNP genotype as a ‘genetic instrument’ operates in a subtly different manner between the different approaches. In the exploratory approaches (SEM, BUF, DEAL, and BNLEARN), the SNP provides information that can be used to help orient the causal direction between the proposed mediator and outcome. In MR, the SNP is instead used as a *surrogate* for the mediator, in order to estimate the mediator's causal effect on the outcome, under the assumption that the SNP associates with outcome only through that particular intermediate variable. MR and the CIT are thus not appropriate for an exploratory analysis of the range of models considered in our study. However, we consider that MR and the CIT could potentially be useful at a later stage of an analysis, after an initial hypothesis generation exercise has taken place.

In MR, the assumptions are critical but in real life applications it can be difficult to ensure they are suitably satisfied (Richmond, Hemani, Tilling, Smith, & Relton, 2016; Ziegler, Mwambi, & König, 2015). In particular, the assumption that the SNP associates with outcome only through the currently considered intermediate biological variable would seem quite unlikely to be met, in practice, for complex biological systems. As expected, our simulation study confirms that in scenarios when the assumptions are met, MR performs as expected. Similarly, in scenarios where the assumptions are violated, MR suggests spurious causal relationships. We note that a possible solution to this issue has recently been addressed through development of the MR‐Egger method (Bowden, Davey Smith, Haycock, & Burgess, 2016), which uses a weighted median estimator of several genetic variants as the instrumental variable in MR. This method gives consistent estimates even when some of the genetic variables are not valid instrumental variables.

The CIT is specifically designed to test whether a variable mediates the association between (and is the only causal link between) a genetic locus and a quantitative trait. It is more flexible than MR because it does not assume that the genetic variant is chosen specifically to be an instrument for the mediator. Due to the way the test is constructed, the CIT is also immune to problems of pleiotropy and reverse confounding. As a result, this method can easily be applied in a model selection context when the aim is to rank many different mediators. However, the CIT does not have a framework for allowing model selection between more complex network structures.

In the initial simulation scenarios we considered, both MR and CIT performed well when their assumptions were satisfied, with the existence of a causal link between mediator and outcome identified 100% of the time under scenario (b). However, one might expect that the performance of both methods would deteriorate when the relationships between the variables (either between instrument *G* and mediator *X* or between mediator *X* and outcome *Y*) are less strong, and, indeed, that is what we find (Supporting Information Fig. S1), with MR achieving overall higher power than the CIT in this situation.

The other four methods for causal inference that we considered allow a much wider range of potential causal structures. For simple causal scenarios, with no unmeasured environmental effects, the performance of these four methods at disentangling the true causal relationships in simulated data was consistently good. In these situations, no method stands out as being uniformly the best. However, we note that for DEAL, poor specification of the imaginary sample size parameter can lead to over‐parameterised models, even in very simple cases. For more complex scenarios, with an unmeasured environmental effect, the performance of the methods at identifying the true causal structure was less accurate. In these scenarios, DEAL and BNLEARN tend to suggest models that contain the correct underlying causal structure but with the addition of extra edges. This is not surprising, as, by adding an environmental effect in our simulated data sets, we have induced further correlation between variables. We observed that in certain situations, SEM and BUF suggest spurious causal relationships in the presence of an environmental effect. For example in scenario (h), SEM mistakenly suggests that the effect of the SNP is mediated through another variable.

A limitation of our simulation study is that we only consider the simplistic case where we have three measured variables. It is important to consider how these methods would scale to larger numbers of variables, as would be encountered in practice in real omics data sets. MR and the CIT do not naturally have a framework for incorporating more variables in the analysis. However, there has been much interest in trying to extend MR to more complex scenarios, see Smith and Hemani (2014) for a review. For example, network MR (Burgess, Daniel, Butterworth, Thompson, & EPIC‐InterAct Consortium, 2015) can consider more complex scenarios than the standard MR framework. More recently, Yazdani, Yazdani, Samiei, and Boerwinkle (2016b) have proposed the GDAG (granularity directed acyclie graph) algorithm which uses a principal component approach to capture information from multiple SNPs across the genome before taking these principal components forward to use in a causal inference scheme (Yazdani, Yazdani, & Boerwinkle, 2016a; Yazdani, Yazdani, Samiei, & Boerwinkle, 2016c; Yazdani, Yazdani, Saniei, & Boerwinkle, 2016d).

An attraction of SEM is that it can handle very complex models with large numbers of variables. However, the user is required to specify precisely which models to test, while making sure these models are not over‐parameterised. If the number of variables was very large, it could potentially become very time consuming for the user specify the full set of models. BUF can very easily incorporate many more phenotypes in the analysis, with the full space of partitions being considered automatically. However, because the end result of a BUF analysis is to partition variables into three groups reflecting their association with the genetic variant, this would only give a very partial insight into the overall causal structure. The Bayesian network methods can incorporate larger numbers of variables relatively seamlessly, using efficient algorithms to step through the possible space of models. These approaches thus arguably represent the most natural class of methods for use with larger numbers of variables, as are routinely starting to be generated using omics technologies. Given their generally good performance when applied to the three‐variable situation considered here, we consider these approaches the most promising avenue for further investigation in application to more complex, multi‐omics data sets.

## Supporting information

Supporting Information Figure S1. Results of applying MR and CIT to data sets simulated under scenario (b). Figure S2: Results of applying SEM to simulated data sets. Figure S3: Results of applying BUF to simulated data sets. Figure S4: Results of applying DEAL to simulated data sets. Figure S5: Results of applying BNLEARN to simulated data sets. Figure S6: Results showing the effect of changing the imaginary sample size (ISS) for the DEAL implementation.
