# Supplementary material for: A comparison of methods for inferring causal relationships between genotype and phenotype using additional biological measurements
Source: Genet Epidemiol. 2017 Jul 10;41(7):577–86. doi: 10.1002/gepi.22061 (PMC5655748; doi:10.1002/gepi.22061)
Supplement: Supplementary file 1 — Supporting Information Figure S1. Results of applying MR and CIT to data sets simulated under scenario (b). Figure S2: Results of applying SEM to simulated data sets. Figure S3: Results of applying BUF to simulated data sets. Figure S4: Results of applying DEAL to simulated data sets. Figure S5: Results of applying BNLEARN to simulated data sets. Figure S6: Results showing the effect of changing the imaginary sample size (ISS) for the DEAL implementation. [file GEPI-41-577-s001.pdf]

Supporting Material: "A comparison of methods for inferring causal relationships between genotype and phenotype using additional biological measurements"

Supplementary Text

Here we present a more detailed description of the statistical methods used in our analysis.

Mendelian randomisation (MR)

The idea behind MR can be dated back to the 1980s [Katan, 1986]. This method can be used to infer the direction of causality between two variables in the presence of confounders. Given observed data on two quantitative (continuous) variables, a genetic variant is carefully selected such that it acts as an instrument for one of the variables. This provides an 'anchor' from which to determine the direction of the causal relationship [Smith and Ebrahim, 2003; Lawlor et al., 2008]. The idea can be thought of in terms of a randomised control trial [Thanassoulis and O'Donnell, 2009] with the randomisation element happening when alleles are randomly allocated to individuals at meiosis. MR has been used extensively in the genetic epidemiology literature to establish causality between an observable intermediate trait and a phenotypic outcome of interest.

In our set-up, MR can be used to identify a causal relationship between X and Y in the presence of a confounder E. This is akin to the situation displayed in model (i) of Figure 1. Crucially, if this were a true MR analysis, the genetic variant G would need to have been chosen to be a valid instrument for X. To be a valid instrument, G must be (1) associated with X, (2) independent of E and (3) associated with Y only via its association with X [Bowden and Turkington, 1984; Didelez and Sheehan, 2007]. If these conditions are satisfied, and assuming the associations are linear, it can be shown that the causal effect from X to Y is

$$\hat{\beta}_{X,Y} = \frac{\hat{\beta}_{G,Y}}{\hat{\beta}_{G,X}}$$

where $\hat{\beta}_{G,X}$ is the regression coefficient resulting from a regression of X on G and $\hat{\beta}_{G,Y}$ is the regression coefficient resulting from a regression of G on Y [Didelez and Sheehan, 2007; Thomas and Conti, 2004]. These coefficients may be estimated via linear regression [Burgess et al., 2011] or Bayesian methods [McKeigue et al., 2010].

The causal inference test (CIT)

The CIT [Millstein et al., 2009] is designed to establish whether a measured variable acts as a mediator (and is the only causal link) between a genetic factor and a phenotype. For example, this test could be used to infer if model (b) of Figure 1 was the true causal scenario, given observed data on the three variables. Unlike MR, the CIT does not suffer due to problems of pleiotropy and reverse causation. Consequently, the CIT can be used where there exists less prior knowledge about the direction of the hypothesized causal relationship.

The methodology can be summarised by stating that a causal link $G \rightarrow X \rightarrow Y$ exists if it can be shown that the following four conditions, derived from the Causal Equivalence Theorem [Chen et al., 2007], are met:

1. G is associated with Y.

2. G is associated with X given Y.

3. X is associated with Y given G.

4. G is independent of Y given X.

The first three of these conditions are straightforward to test, however, testing the fourth condition is more involved and amounts to an equivalence testing problem. An overall p-value for the test is taken to be the maximum of the p-values from the four individual tests, this is equivalent to the intersection-union test [Berger and Hsu, 1996]. The CIT can easily be implemented in R using the package `cit` [Millstein, 2016].

Structural equation modelling (SEM)

Structural equation modelling (SEM) has a vast literature spanning several disciplines [Bollen, 1989], however its origins can be traced back to the methods of path analysis [Wright, 1921; Pearl, 2000] and confirmatory factor analysis [Bollen, 1989; Jöreskog, 1967]. SEM is typically used as a confirmatory tool, to decide whether or not a hypothesised model is valid, rather than to explore possible models. However, for the purposes of our study, we consider the performance of SEM as an exploratory tool to ascertain the most plausible causal model (from a set of possible models) given the observed data.

In a SEM analysis, the model is represented using a path diagram similar to those in Figure 1. Unlike Figure 1, the path diagram has a more formal syntax whereby observed and unobserved variables are distinguished by enclosing them in rectangles and circles/ovals respectively. A single-headed straight arrow between two variables indicates a causal relationship. Given a path diagram, a series of linear equations can be constructed which represent the beliefs encoded in the path diagram. The parameters of the model $\boldsymbol{\theta}$ are then jointly estimated using, for example, maximum likelihood techniques. Using these parameter estimates, the predicted covariance matrix $\Sigma(\boldsymbol{\theta})$ is constructed. This covariance matrix represents the form the covariance matrix S would take if the model were true. The predicted covariance matrix is then compared to the sample covariance matrix to assess whether the two are consistent with one another. There are many possible functions that can be used to evaluate the fit of a SEM model. We choose to use the most popular, that is the maximum likelihood function

$$F_{ML} = log|\Sigma(\boldsymbol{\theta})| + tr(S\Sigma^{-1}(\boldsymbol{\theta})) - \log|S| - n$$

which has asymptotic distribution

$$(N-1)F_{ML} \sim \chi^2.$$

Here, N is the number of subjects in the data set and n represents the number of variables in

the model. There exist many criteria through which competing models can be compared. We choose to use the Bayesian information criterion (BIC) [Schwarz, 1978]. We used the R package `sem` [Fox et al., 2015] for our analyses, however, other packages such as `lavaan` [Rosseel, 2012] will produce equivalent results.

Bayesian Unified Framework (BUF)

The Bayesian Unified Framework (BUF) approach [Stephens, 2013] was developed as a method for analysing multivariate phenotypes, for example in GWAS. However, we consider this a potentially interesting method which can be used to learn about the underlying causal structure in data. We give a very brief overview of an implementation of the method below, and refer the reader to the original text [Stephens, 2013] for technical details.

Let $\boldsymbol{\mathcal{Y}}$ represent a matrix of observed phenotypes, where columns represent phenotypes and rows represent individuals. For our analysis we have two phenotypes, X and Y, and thus $\boldsymbol{\mathcal{Y}} = (X, Y)$. Assuming a single genetic variant G, the genetic data is contained in a vector $\boldsymbol{g}$. The method aims to partition the variables contained in $\boldsymbol{\mathcal{Y}}$ into

$$\gamma = (U, D, I)$$

such that variables contained in U are unassociated with G, variables contained in D are directly associated and variables in I are indirectly associated with G. Clearly, given these partitions, it is possible to reconstruct path diagrams such as those in Figure 1. In our framework, any variable classified as being in D would have an arrow going directly to it from G, and any variable classified as I would have a path to it from G via another variable. When a variable is classified as U, this would represent the fact that neither a direct nor an indirect path from G to the variable exists.

For each possible partition of variables γ, there exists a probability model $p_\gamma(\boldsymbol{\mathcal{Y}}|\boldsymbol{g})$. Under the assumption that, for each genotype class, the outcomes $\boldsymbol{\mathcal{Y}}$ are multivariate normal, Bayesian multivariate regression can be used to specify these distributions. In the BUF framework, an exhaustive set of partitions are evaluated and the support for each model is

given by the Bayes Factor:

$$BF_\gamma = \frac{p_\gamma(\boldsymbol{\mathcal{Y}}|\boldsymbol{g})}{p_0(\boldsymbol{\mathcal{Y}})}$$

where $p_0(\boldsymbol{\mathcal{Y}})$ represents the global null, that is, the model where all variables are unassociated with G. Under certain assumptions, $p_\gamma(\boldsymbol{\mathcal{Y}}|\boldsymbol{g})$ can be shown to factorise in such a way that renders the above equation relatively easy to evaluate. In our simulations, the partition of variables with the highest Bayes Factor is considered to be the partition that is most plausible.

Bayesian networks

The problem of inferring causal structure amongst a set of variables naturally lends itself to the Bayesian network framework. Formally, Bayesian networks are graphical representations of the probabilistic relationships between random variables. This graphical representation takes the form of a graph in which nodes represent variables and directed edges represent arrows. Since cycles within the graphs are not permitted, these are known as directed acyclic graphs (DAGs). The diagrams in Figure 1 can be thought of as DAGs.

In any DAG, two nodes are conditionally independent if no edge between them exists. This means that the joint (or global) probability distribution (JPD) of a Bayesian network can be factorised into conditional probability distributions (local distributions). For example, for model (b) in Figure 1, the JPD can be factorised as follows

$$P(G, X, Y) = P(G)P(X|G)P(Y|X).$$

This property is very useful since, in its factorised form, the JPD can be parameterised with far fewer parameters, this is especially useful when the network becomes large. For Bayesian networks with discrete variables, a sensible choice for the JPD is the multinomial distribution. In this case the local distributions are also all multinomial. When variables are continuous, it is typical that the multivariate normal distribution is used. These networks are known as Gaussian Bayesian Networks and in this case the local distributions are univariate

normal. For networks containing both discrete and continuous variables the procedure is more complex. However, if it is assumed that discrete nodes may not have continuous parents, the local distributions can be specified such that the JPD factorises into a part that is discrete and a part that is mixed [Böttcher, 2001]. The JPD is then conditional Gaussian.

Our interest lies in learning the structure of Bayesian networks given observed data. We proceed by calculating for each candidate Bayesian network a score (related to the likelihood of the data under that network) and consider the network with the highest score to be the one that is most plausible. Given the small number of variables we consider, evaluating the score for every possible Bayesian network is not time consuming. However, for much larger networks, efficient algorithms can be used to move through the space of possible Bayesian networks whilst maximizing the network score [Larranaga et al., 1997].

We choose to focus on two R packages for learning Bayesian networks: `deal` [Bottcher and Dethlefsen., 2013] and `bnlearn` [Scutari, 2010] referring to these implementations as DEAL and BNLEARN respectively. Although there exist many other packages [Kalisch et al., 2012; Balov and Salzman, 2012] for learning Bayesian networks, the two we consider are the only two (as far as we are aware) that can handle both discrete and continuous variables. In the DEAL implementation, the network score used is the Bayesian Dirichlet equivalent score [Böttcher, 2001; Geiger and Heckerman, 1994; Heckerman et al., 1995]. In the BNLEARN implementation, the network score used is the Bayesian information criteria (BIC) [Schwarz, 1978]; this has the structure of a penalised log-likelihood.

References

Balov N and Salzman P. 2012. Catnet: categorical Bayesian network inference. *R package version* 1.

Berger RL and Hsu JC. 1996. Bioequivalence trials, intersection-union tests and equivalence confidence sets. *Statistical Science* 11: 283–319.

Bollen KA. 1989. *Structural equations with latent variables.* John Wiley & Sons.

Böttcher SG. 2001. Learning Bayesian networks with mixed variables. *Artificial Intelligence and Statistics, January 2001, Key West, Florida* .

Bottcher SG and Dethlefsen C. 2013. *deal: Learning Bayesian Networks with Mixed Variables.* R package version 1.2-37.

Bowden RJ and Turkington DA. 1984. *Instrumental variables.* Cambridge University Press.

Burgess S, Thompson SG and CRP CHD Genetics Collaboration. 2011. Avoiding bias from weak instruments in Mendelian randomization studies. *International Journal of Epidemiology* 40: 755–764.

Chen LS, Emmert-Streib F and Storey JD. 2007. Harnessing naturally randomized transcription to infer regulatory relationships among genes. *Genome Biology* 8: R219.

Didelez V and Sheehan N. 2007. Mendelian randomization as an instrumental variable approach to causal inference. *Statistical Methods in Medical Research* 16: 309–330.

Fox J, Nie Z and Byrnes J. 2015. *sem: Structural Equation Models.* R package version 3.1-6.

Geiger D and Heckerman D. 1994. Learning gaussian networks. In *Proceedings of the Tenth International Conference on Uncertainty in Artificial Intelligence*, pp. 235–243. Morgan Kaufmann Publishers Inc.

Heckerman D, Geiger D and Chickering DM. 1995. Learning Bayesian networks: The combination of knowledge and statistical data. *Machine Learning* 20: 197–243.

Jöreskog KG. 1967. A general approach to confirmatory maximum likelihood factor analysis. *ETS Research Bulletin Series* 1967: 183–202.

Kalisch M, Mächler M, Colombo D, Maathuis MH and Bühlmann P. 2012. Causal inference using graphical models with the R package pcalg. *Journal of Statistical Software* 47: 1–26.

Katan M. 1986. Apolipoprotein E isoforms, serum cholesterol, and cancer. *Lancet (London, England)* 1: 507.

Larranaga P, Sierra B, Gallego M, Michelena M and Picaza J. 1997. Learning Bayesian networks by genetic algorithms: a case study in the prediction of survival in malignant skin melanoma. *Artificial Intelligence in Medicine* pp. 261–272.

Lawlor DA, Harbord RM, Sterne JA, Timpson N and Davey Smith G. 2008. Mendelian randomization: using genes as instruments for making causal inferences in epidemiology. *Statistics in Medicine* 27: 1133–1163.

McKeigue PM, Campbell H, Wild S, Vitart V, Hayward C, Rudan I, Wright AF and Wilson JF. 2010. Bayesian methods for instrumental variable analysis with genetic instruments (Mendelian randomization): example with urate transporter SLC2A9 as an instrumental variable for effect of urate levels on metabolic syndrome. *International Journal of Epidemiology* 39: 907–918.

Millstein J. 2016. *cit: Causal Inference Test.* R package version 2.0.

Millstein J, Zhang B, Zhu J and Schadt EE. 2009. Disentangling molecular relationships with a causal inference test. *BMC Genetics* 10: 23.

Pearl J. 2000. *Causality: Models, reasoning, and inference.* Cambridge University Press.

Rosseel Y. 2012. lavaan: An R package for structural equation modeling. *Journal of Statistical Software* 48: 1–36.

Schwarz G. 1978. Estimating the dimension of a model. *The Annals of Statistics* 6: 461–464.

Scutari M. 2010. Learning Bayesian networks with the bnlearn R package. *Journal of Statistical Software* 35: 1–22.

Smith GD and Ebrahim S. 2003. Mendelian randomization: can genetic epidemiology contribute to understanding environmental determinants of disease? *International Journal of Epidemiology* 32: 1–22.

Stephens M. 2013. A unified framework for association analysis with multiple related phenotypes. *PloS One* 8: e65245.

Thanassoulis G and O'Donnell CJ. 2009. Mendelian randomization: nature's randomized trial in the post–genome era. *Journal of the American Medical Association* 301: 2386–2388.

Thomas DC and Conti DV. 2004. Commentary: the concept of 'Mendelian Randomization'. *International Journal of Epidemiology* 33: 21–25.

Wright S. 1921. Correlation and causation. *Journal of Agricultural Research* 20: 557–585.

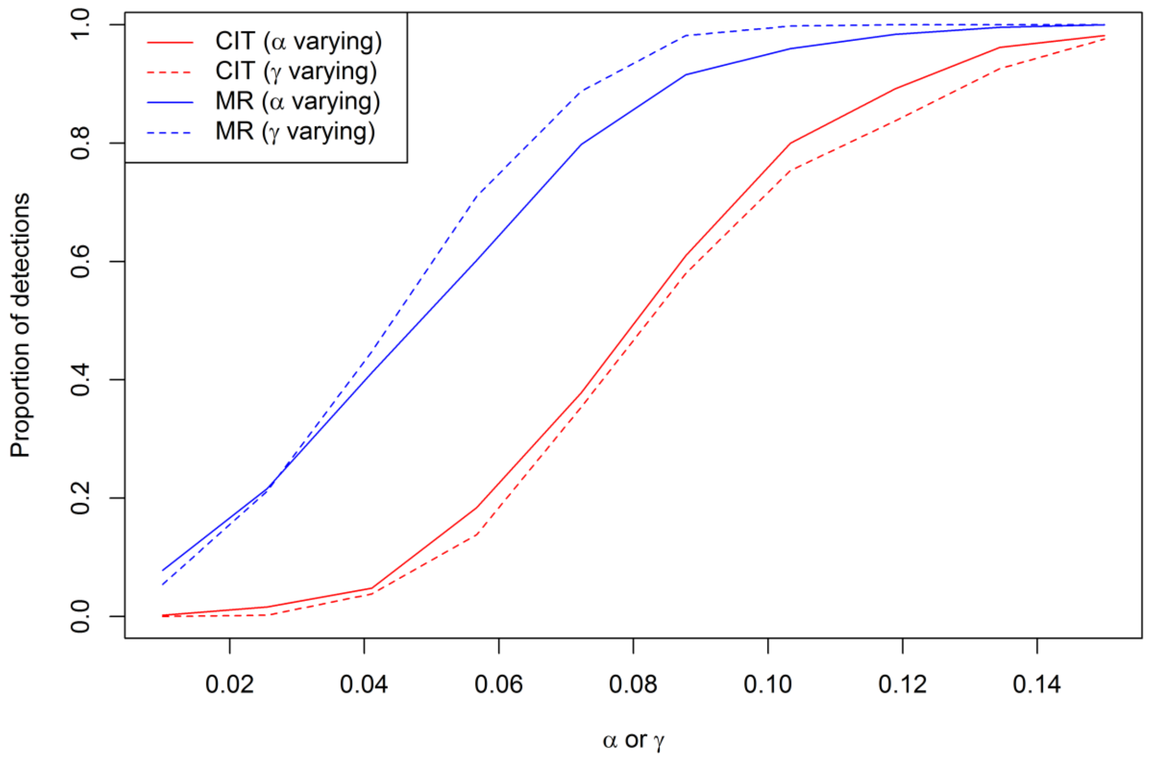

Supplementary Figure 1: Results of applying MR and CIT to datasets simulated under scenario (b). Solid lines represent scenarios where $\propto$ varies (and γ is fixed at one). Dotted lines represent scenarios where γ varies (and $\propto$ is fixed at one).

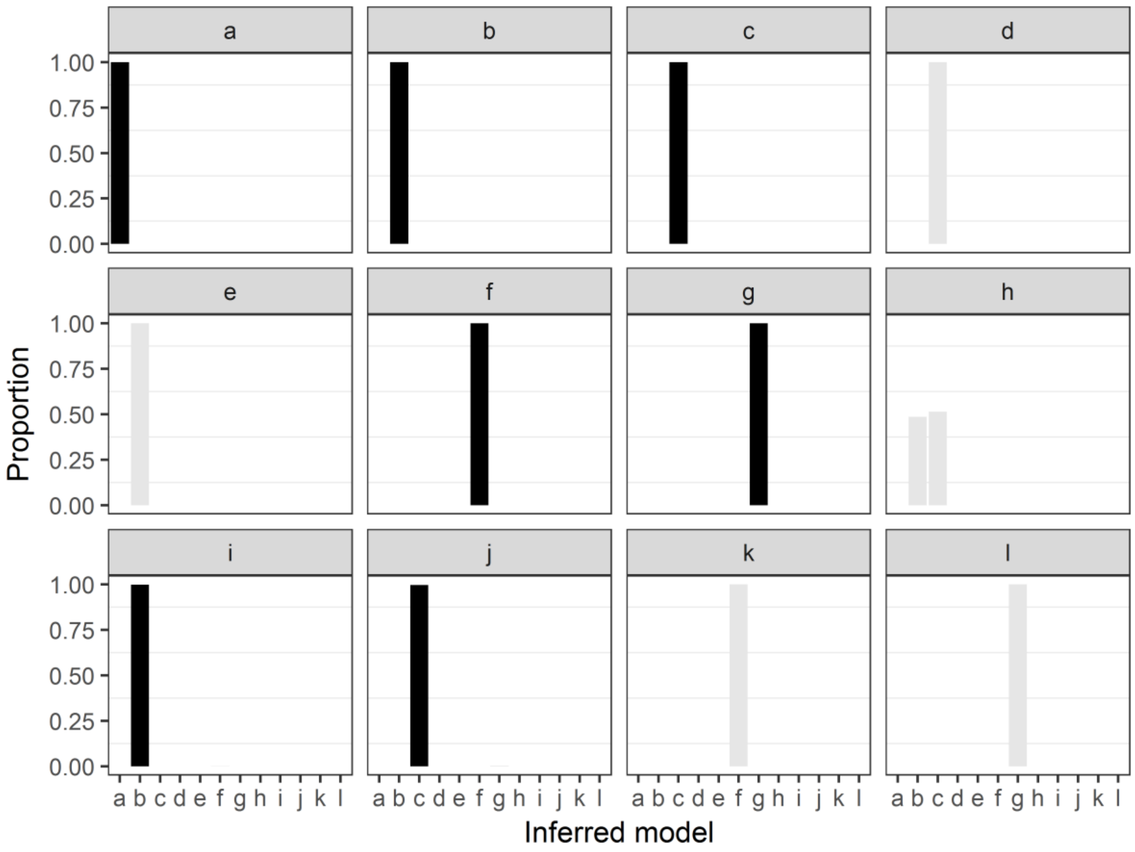

Supplementary Figure 2: Results of applying SEM to simulated datasets. Each panel refers to results of inference on data simulated under the model given in the heading. Individual panels show the proportion of time (y-axis) that each model (x-axis) was selected as being the most likely causal model. Black and grey represent `true' and `false' identifications respectively of the underlying causal model.

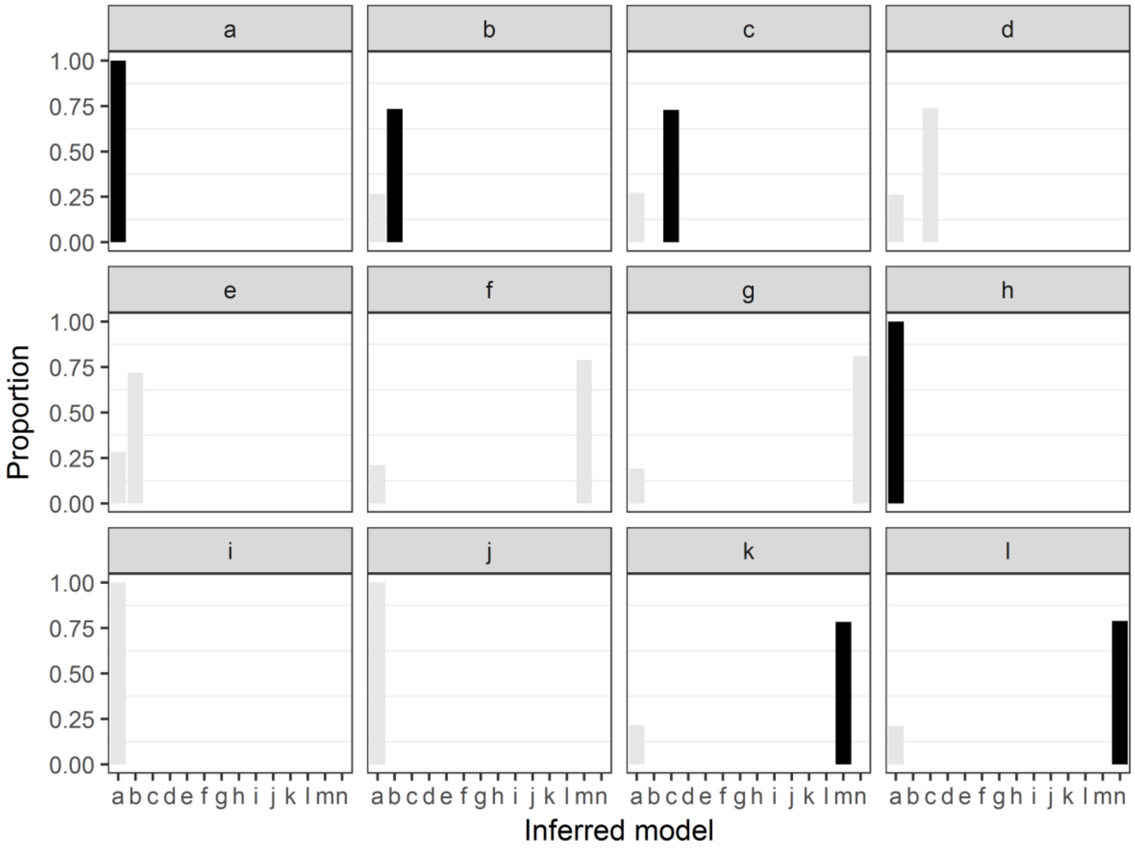

Supplementary Figure 3: Results of applying BUF to simulated datasets. Each panel refers to results of inference on data simulated under the model given in the heading. Individual panels show the proportion of time (y-axis) that each model (x-axis) was selected as being the most likely causal model. Black and grey represent `true' and `false' identifications respectively of the underlying causal model. Scenario *m* represents the model where there is a single arrow G → X and similarly *n* which represents G → Y.

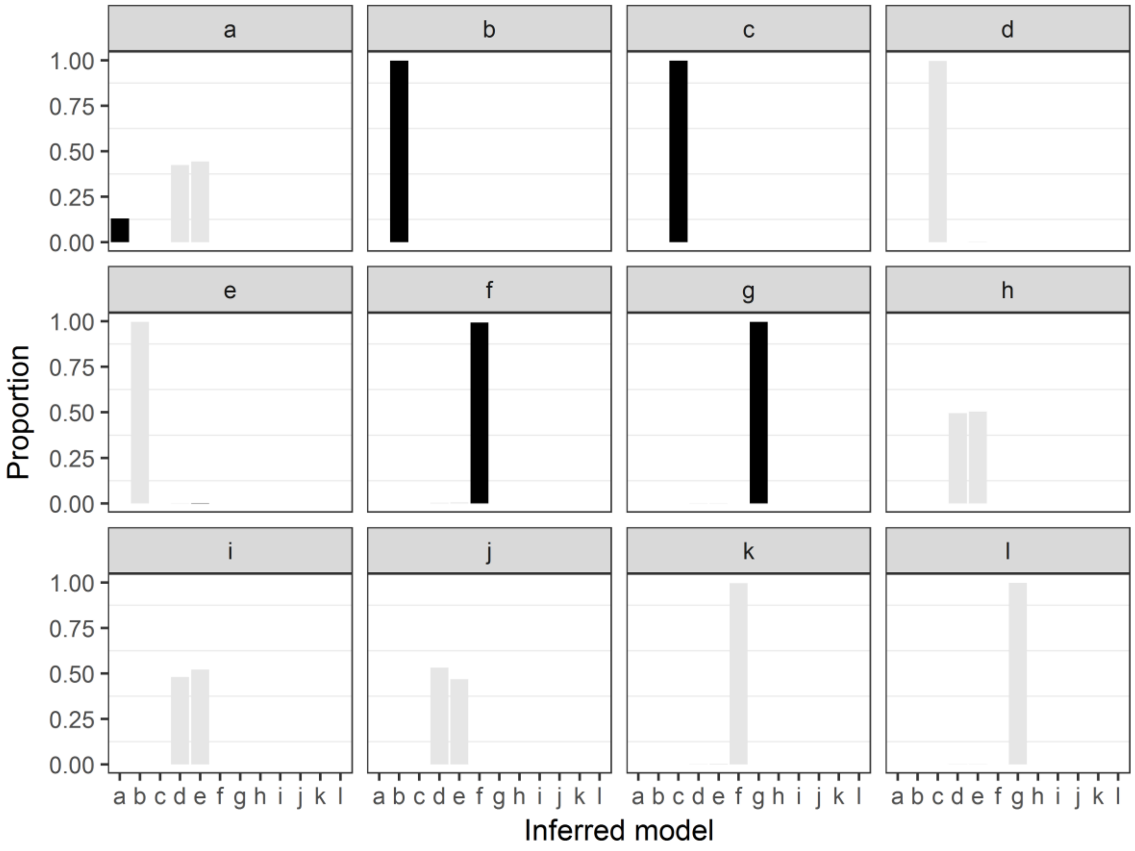

Supplementary Figure 4: Results of applying DEAL to simulated datasets. Each panel refers to results of inference on data simulated under the model given in the heading. Individual panels show the proportion of time (y-axis) that each model (x-axis) was selected as being the most likely causal model. Black and grey represent `true' and `false' identifications respectively of the underlying causal model.

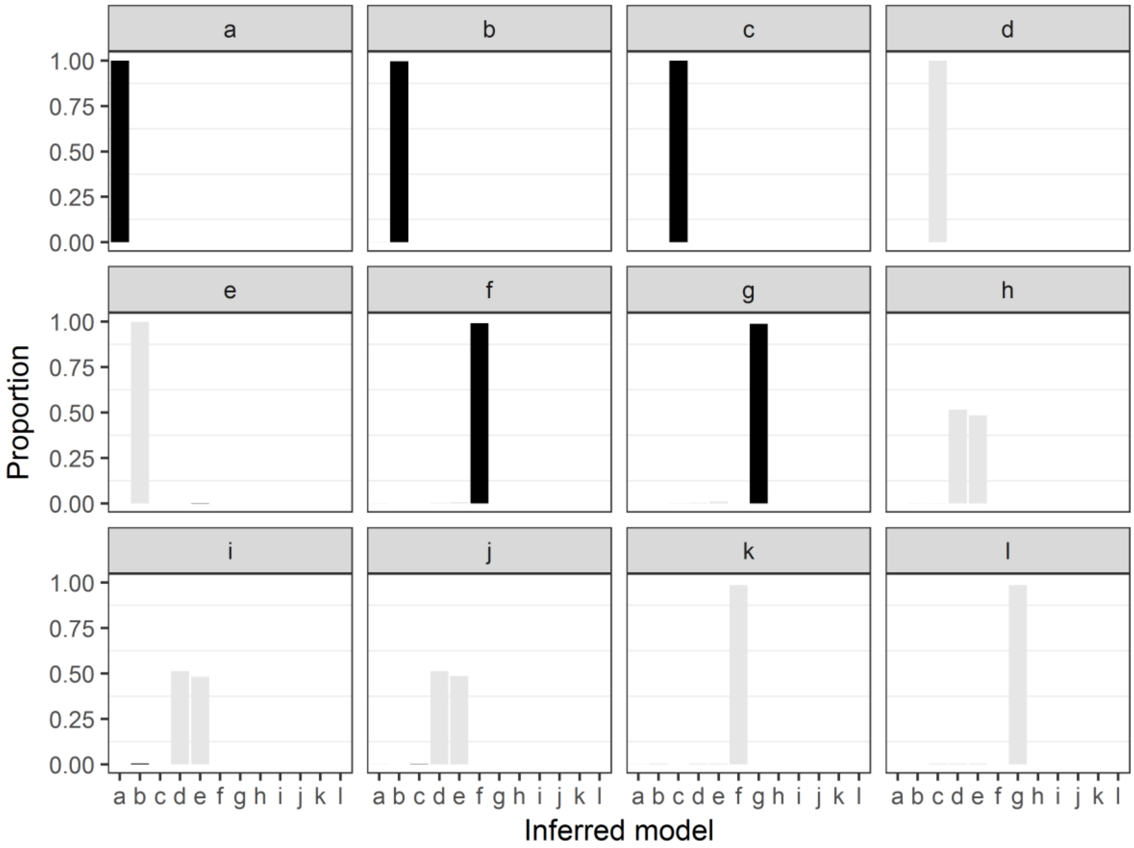

Supplementary Figure 5: Results of applying BNLEARN to simulated datasets. Each panel refers to results of inference on data simulated under the model given in the heading. Individual panels show the proportion of time (y-axis) that each model (x-axis) was selected as being the most likely causal model. Black and grey represent `true' and `false' identifications respectively of the underlying causal model.

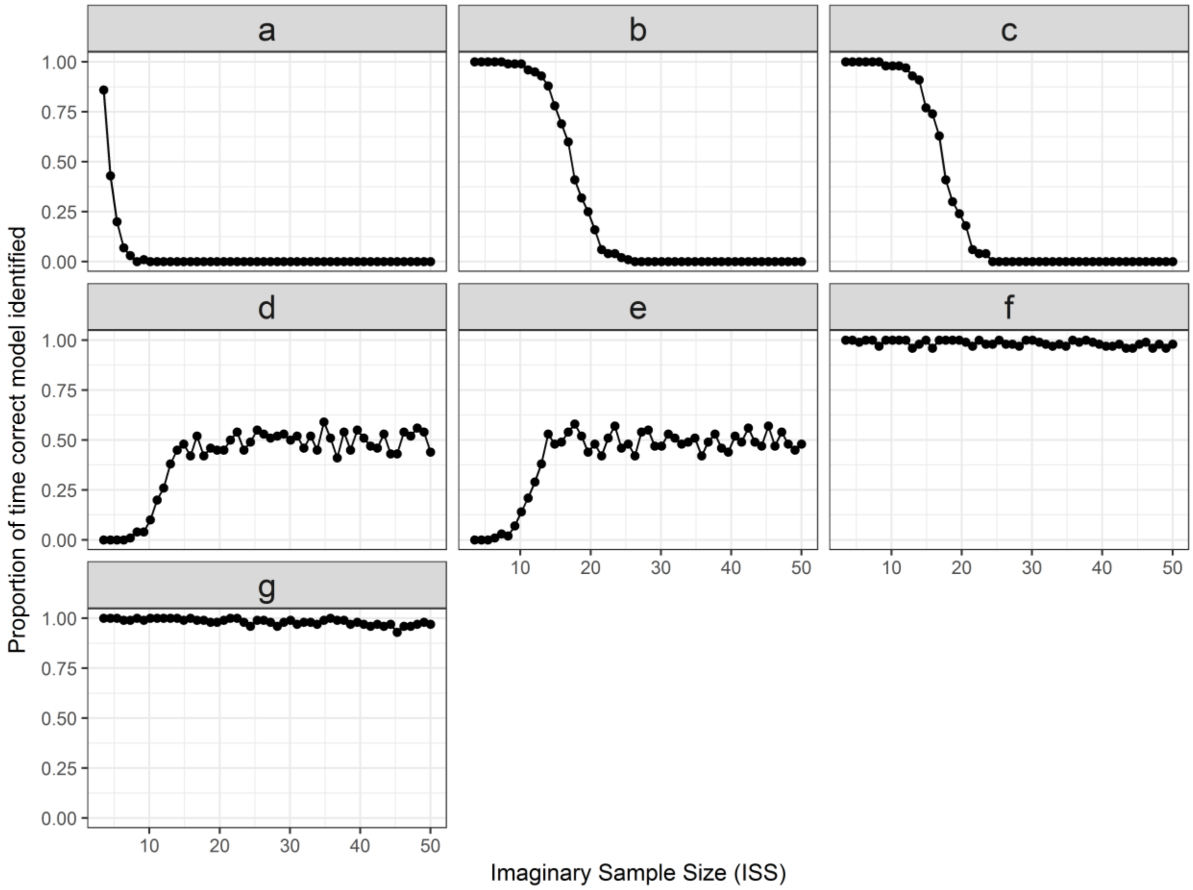

Supplementary Figure 6: Results showing the effect of changing the imaginary sample size (ISS) for the DEAL implementation. Each panel refers to data simulated under different models. The graphs show how the proportion of times the correct model is identified (y-axis) changes with ISS (x-axis).
